# Potential Usage of Edible Mushrooms and Their Residues to Retrieve Valuable Supplies for Industrial Applications

**DOI:** 10.3390/jof7060427

**Published:** 2021-05-28

**Authors:** Harsh Kumar, Kanchan Bhardwaj, Ruchi Sharma, Eugenie Nepovimova, Natália Cruz-Martins, Daljeet Singh Dhanjal, Reena Singh, Chirag Chopra, Rachna Verma, Kamel A. Abd-Elsalam, Ashwani Tapwal, Kamil Musilek, Dinesh Kumar, Kamil Kuča

**Affiliations:** 1 School of Bioengineering & Food Technology, Shoolini University of Biotechnology and Management Sciences, Solan 173229, India; microharshs@gmail.com (H.K.); mails4sharmaruchi@gmail.com (R.S.); 2 School of Biological and Environmental Sciences, Shoolini University of Biotechnology and Management Sciences, Solan 173229, India; kanchankannu1992@gmail.com (K.B.); rachnaverma@shooliniuniversity.com (R.V.); 3 Department of Chemistry, Faculty of Science, University of Hradec Kralove, 50003 Hradec Kralove, Czech Republic; eugenie.nepovimova@uhk.cz (E.N.); kamil.musilek@uhk.cz (K.M.); 4 Faculty of Medicine, University of Porto, Alameda Prof. Hernani Monteiro, 4200-319 Porto, Portugal; ncmartins@med.up.pt; 5 Institute for Research and Innovation in Health (i3S), University of Porto, 4200-135 Porto, Portugal; 6 Laboratory of Neuropsychophysiology, Faculty of Psychology and Education Sciences, University of Porto, 4200-135 Porto, Portugal; 7 School of Bioengineering and Biosciences, Lovely Professional University, Phagwara, Punjab 144411, India; daljeetdhanjal92@gmail.com (D.S.D.); reena.19408@lpu.co.in (R.S.); chirag.18298@lpu.co.in (C.C.); 8 Agricultural Research Center (ARC), Plant Pathology Research Institute, Giza 12619, Egypt; kamelabdelsalam@gmail.com; 9 Forest Protection Division, Himalayan Forest Research Institute, Shimla 171013, India; ashwanitapwal@gmail.com; 10 Biomedical Research Center, University Hospital Hradec Kralove, 50005 Hradec Kralove, Czech Republic

**Keywords:** edible mushrooms, waste valorization, food products, industrial applications

## Abstract

Currently, the food and agricultural sectors are concerned about environmental problems caused by raw material waste, and they are looking for strategies to reduce the growing amount of waste disposal. Now, approaches are being explored that could increment and provide value-added products from agricultural waste to contribute to the circular economy and environmental protection. Edible mushrooms have been globally appreciated for their medicinal properties and nutritional value, but during the mushroom production process nearly one-fifth of the mushroom gets wasted. Therefore, improper disposal of mushrooms and untreated residues can cause fungal disease. The residues of edible mushrooms, being rich in sterols, vitamin D2, amino acids, and polysaccharides, among others, makes it underutilized waste. Most of the published literature has primarily focused on the isolation of bioactive components of these edible mushrooms; however, utilization of waste or edible mushrooms themselves, for the production of value-added products, has remained an overlooked area. Waste of edible mushrooms also represents a disposal problem, but they are a rich source of important compounds, owing to their nutritional and functional properties. Researchers have started exploiting edible mushroom by-products/waste for value-added goods with applications in diverse fields. Bioactive compounds obtained from edible mushrooms are being used in media production and skincare formulations. Furthermore, diverse applications from edible mushrooms are also being explored, including the synthesis of biosorbent, biochar, edible films/coating, probiotics, nanoparticles and cosmetic products. The primary intent of this review is to summarize the information related to edible mushrooms and their valorization in developing value-added products with industrial applications.

## 1. Introduction

Mushrooms have long been stated as a gourmet food, especially for its subtle flavor and taste, and they have been regarded as a culinary wonder by humankind. There are 2000 different mushrooms, out of which 25 are usually consumed as food, and only a few are commercially grown [1]. Mushrooms are also used as nutraceutical foods for their high functional and nutritional value. Moreover, they have gained considerable attention due to their economic importance as well as organoleptic and medicinal properties [2,3]. It is not easy to differentiate between medicinal and edible mushrooms, as few medicinal mushrooms are edible, and many common edible mushrooms have therapeutic potential [4]. The most widely cultivated mushroom is *Agaricus bisporus,* followed by *Flammulina velutipes, Lentinus edodes* and *Pleurotus* spp. The crude protein content of edible mushrooms is usually high, but it varies greatly and is affected by factors such as species and stage of development of the mushroom [5]. The free amino acid level of mushrooms is usually low, ranging from 7.14 to 12.3 mg g^−1^ in dry edible mushrooms, and contributes to the main flavor properties of mushrooms [6]. The essential amino acid profiles of mushrooms reveal that the proteins are deficient in sulfur-containing amino acids, including methionine and cysteine. However, these edible mushrooms are comparatively rich in threonine and valine. Several vitamins such as folates, niacin and riboflavin are found in abundance in cultivated mushrooms. Mushrooms have a higher vitamin B2 content compared to most vegetables, making them a good vitamin source [7]. The bioavailable form of folate in mushrooms is folic acid [8]. Cultivated mushrooms also comprise vitamin B1 and vitamin C in small quantities and traces of vitamin B12 [7]. Edible mushrooms contain a low amount of total soluble sugars, whereas oligosaccharides are found abundantly [9]. The carbohydrate content of edible mushrooms ranges from 35 to 70% by dry weight and varies from species to species. The fatty acid level ranges from 2 to 8% in mushrooms. Additionally, polyunsaturated fatty acids account for ≥75% of total fatty acids, in contrast to saturated fatty acids, and palmitic acid is the major saturated fatty acid [10].

**Figure 1:**
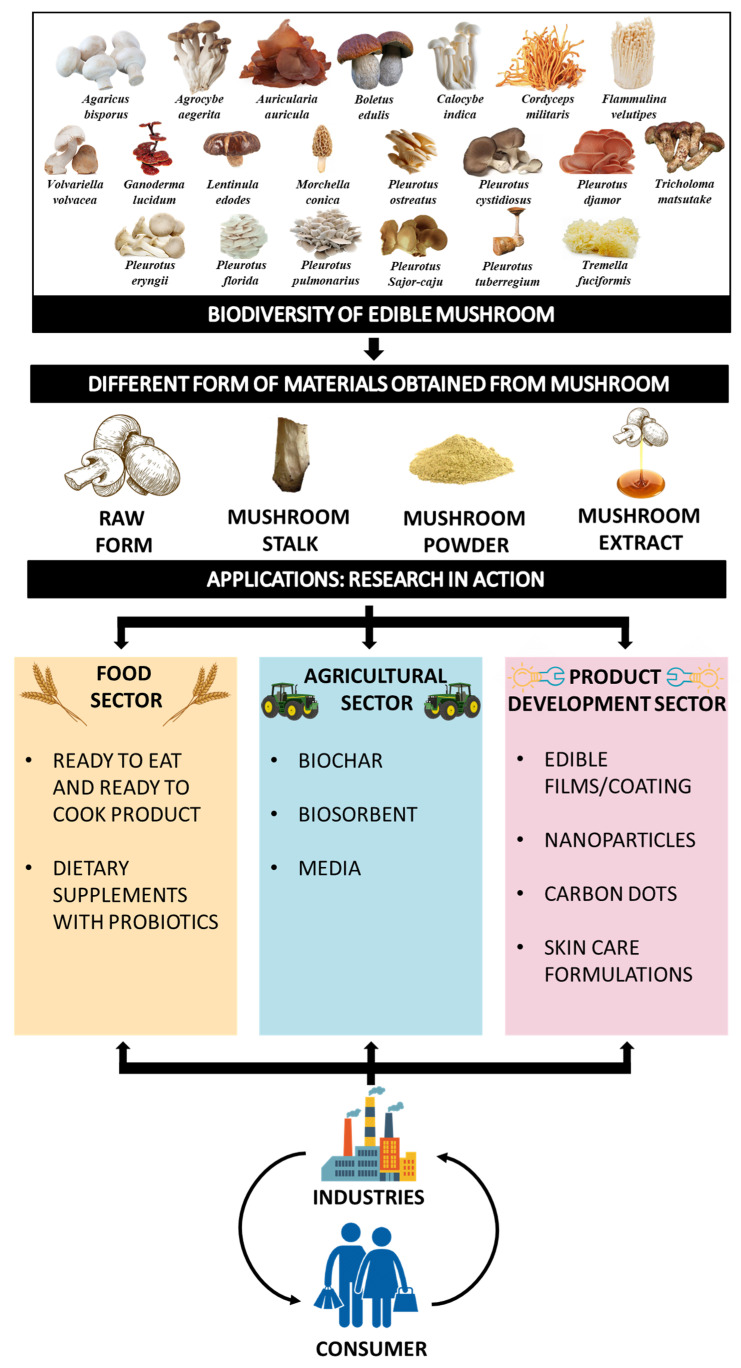
Utilization of edible mushrooms and their residues in novel industrial products.

Many by-products (caps, stipes, spent mushroom substrate) are produced during mushroom production, which cause environmental pollution and increase industry management costs [11]. Spent mushroom substrate (SMS) encompasses extracellular enzymes, fungal mycelia and other substances [12]. The circular economy concept of industrial ecology is regarded as the leading principle for developing new products by using waste as a raw material [13]. From economic and environmental perspectives, the waste produced during mushroom production often leads to massive damage to valuable organic constituents and raises severe management complications. Thus, there is a need to exploit mushroom residues to extract valuable compounds that could be used in different industries, such as food, cosmetics, agricultural and textile industries, as depicted in Figure 1. The current review aims to summarize information related to edible mushrooms and discuss the utilization of edible mushrooms and their residues as a valuable good for future industrial applications.

## 2. Edible Mushrooms Fortified in Ready-to-Eat and Ready-to-Cook Foods

As the lifestyle of people is changing dramatically (due to liberalization policies, dual incomes, separate living of couples, innovative kitchen applications, media proliferation, etc.), the demand for convenient and health-promoting food is also increasing. Nowadays, people prefer fast and simple cooking methods instead of spending a long time in the kitchen [14]. Mushroom powder can be used in the food industry, especially in preparing baked goods (bread, biscuits and cakes) and breakfast cereals. The supplementation of mushroom powder in bakery products substantially increases crude fibers, minerals (calcium, copper, magnesium, manganese, potassium, phosphorus, iron and zinc), proteins and vitamins [15]. These components impart the abilities to fight tumors, lower blood pressure and blood sugar levels, maintain cholesterol levels and improve the immune system to fight against infection [16]. Rathore et al. [17] prepared cookies fortified with *Calocybe indica* mushroom, and the results depicted a decrease in starch hydrolysis and glycemic index. Wheatshiitake noodles enhanced the nutritional profile and reduced the glycemic index of foods [18]. The different food products developed by using mushrooms are listed in Table 1.

## 3. Edible Mushrooms Based Films/Coatings

Edible films/coatings are thin layers applied on the food surface to extend their shelf-life and preserve their features, functionality and properties at a low cost [44]. The mechanical strength and barrier properties of these edible films provide sufficient strength to withstand stress while handling. These films have a promising application in the agricultural, food and pharmaceutical industries. Mushrooms and their residues have many applications in food industries, but significantly fewer studies have been conducted in regards to edible film/coatings. Polysaccharides extracted/derived from edible mushrooms are extensively used in functional foods, pharmaceuticals and nutraceuticals [11]. In this regard, Bilbao-Sainzand his colleagues [45] obtained chitin from mushrooms and transformed it to chitosan.

Moreover, layer-by-layer (LbL) electrostatic deposition is used to prepare edible coatings applied to fruit bars. The application of edible mushroom coatings/films has increased the antioxidant capacity, ascorbic acid content, fungal growth prevention and firmness during storage. Additionally, Du et al. [46] developed edible films using *Flammulina velutipes* polysaccharides, which acted as a barrier to oxygen and water vapor, had the lowest elongation at break values and highest tensile strength for future use in food packaging industries. Table 2 lists some edible films and coatings derived from mushrooms.

## 4. Mushrooms as a Source of Prebiotics for Food Supplementation

The consumption of high dietary fiber food has gained considerable interest owing to its ability to reduce triglycerides and blood cholesterol via the gut microbiome. A diet rich in fibers acts as a substrate for microbes and aids in their proliferation. Thus, microbial digestion products enter the systemic circulation and help in maintaining energy homeostasis [50]. *Pleurotus* spp. (Oyster mushroom) comprises soluble fiber compounds, particularly a small amount of glucans (chitin and galactomannans) and non-starch glucans, favoring the proliferation of *lactobacilli* [51]. Edible mushrooms are stated to have carbohydrates, which help them to act as prebiotics [52]. The supplementation of oyster mushroom and probiotics in poultry feed has been reported to show beneficial, synergetic effects on the immune response, performance and serum lipids in broiler chickens [53]. The blend of prebiotics and probiotics also is beneficial because of the synergistic effect between them [54]. Van Doan et al. [52] conducted a study to determine the effects of dietary supplements *Pleurotus eryngii* (as a prebiotic), Eryngii mushroom and *Lactobacillus plantarum* (as a probiotic), alone as well as in combination, on the innate immune response, growth and protection against *Aeromonas hydrophila.* The results showed stimulation in growth, immunity and disease resistance against *Pangasius bocourti*. Table 3 lists studies of different mushrooms and dietary supplementation with probiotics.

## 5. Edible Mushrooms Based Media

Nowadays, mushroom processing is the primary solid-state fermentation process in fermentation industries [60]. At the commercial level, the processing occurs on a substrate made up of lignocellulose materials (corncobs, sawdust, rye, rice straw and wheat) either alone or in combination with supplements to address nutritional deficiencies [61,62]. For instance, approximately 5 kg of SMS is produced, a by-product of mushroom harvest and cultivation, from 1 kg of mushrooms [63]. The SMS comprises a high amount of residual nutrients, which pollutes the atmosphere if improperly discarded as waste [64,65]. Thus, further treatment and utilization of SMS are essential. Different types of edible mushrooms and their SMS have been used to produce low-cost growth media for various horticultural plants and microorganisms (Table 4).

For tomato seedling production, SMS-derived media from *Agaricus bisporus* and *Pleurotus ostreatus* were used. For pepper seedling production, SMS and a 50% mixture of *Agaricus bisporus* and *Pleurotus ostreatus* were used, while a low dose of SMS derived from *Agaricus bisporus* was reported for courgette seedling production [67]. Owing to the chemical, physical and nutritional characteristics of SMS, Sendi et al. [73] revealed that spent mushroom waste (50%) and peat moss (50%) with nitrogen, phosphorus and potassium fertilizer supplementation were the growth media components that had the greatest potential to aid in Chinese kale growth. SMS derived from *Flammulina velutipes* was appropriate for biopesticide (*Bacillus thuringiensis*) with high toxicity and long larvicidal persistence [69]. *Pleurotus ostreatus* derived SMS, when neutralized with calcium hydroxide, ammonium hydroxide or sodium hydroxide, may be used as a carbon source for *Lactococcus lactis* subsp. *lactis* W28 cultivation [71].

## 6. Edible Mushrooms Derived Biosorbents

Biosorption is a process in which a sorbate reacts with biomass or biomaterial (biosorbent), causing sorbate ions to acclimatize on the biosorbents surface and, as a result, lowering the concentration of sorbate in the solution [44]. This mechanism has gained significant attention among researchers because of its ability to immobilize heavy metals, which can contaminate the water as they are discharged untreated from electroplating, mining industries and metal processing industries. Various processes that explain the mechanism of how these biosorbents function in removing pollutants are expressed by the natural biomass complex compendium. Several functional groups (amides, amine, carboxyl, carbonyl, hydroxyl, sulfonate, sulfhydryl, phosphate and phenolic groups) are attached to these biosorbents to sequestrate contaminants [77,78]. Various studies were done to produce biosorbents from edible mushrooms to remove metal ions and dyes from an aqueous solution, as shown in Table 5.

For the biosorption of lead (II) from aqueous solution, Eliescu et al. [81] compared the biosorbent activity of *Pleurotus ostreatus* biomass derived from spent mushroom substrate and observed Pb(II) sorption parameters. The *Pleurotus ostreatus* spent substrate (POBM) had a higher adsorption potential (85.91 mg g^−1^) than that of the *Pleurotus ostreatus* original spores (PO) (57.73mg g^−1^). Further, the presence of hydroxyl and carbonyl groups in the spent substrate of *Ganodorma lucidum* proved as a biosorbent for cationic dyes (malachite green, methylene blue and safranine) [89]. In another study, the hydroxyl and carboxyl groups in a *Pleurotus eryngii* derived biosorbent were reported to remove nitrate from an aqueous solution [90].

## 7. Edible Mushrooms Derived Biochar

Biochar is a stable, carbon-rich solid prepared by thermochemical decomposition or pyrolysis of organic material at high temperatures in an anaerobic environment [44]. The highly porous structure permits the extraction of humic and fluvic-like substances from biochar [91]. Furthermore, its molecular structure demonstrates high microbial and chemical stability [92], and physical and chemical properties depend on several factors such as the feedstock form, residence time, pyrolysis and furnace temperature [93,94]. A wide range of common raw materials are used as the feedstock, including wood chips, organic wastes, plant residues and poultry manure [95]. The elemental composition of biochar generally includes carbon, nitrogen, hydrogen and, to a lesser extent, K, Ca, Na and Mg [96]. Biochar is a polar or non-polar material with a high specific surface area and good affinity towards inorganic ions such as phosphate, nitrate and heavy metal ions [97,98]. Different studies have reported on biochar production from a variety of edible mushrooms and their spent substrates (Table 6).

Wu et al. [99] investigated how inorganic mineral-induced alkalinity in biochars could facilitate Pb(II) removal by forming Pb(II) precipitate. The X-ray diffraction (XRD) analysis revealed that the spent mushroom substrate (SMS)-derived biochars contained Ca_3_(PO_4_)_2_, CaCO_3_ and inorganic anions (CO_3_^2−^, SO_4_^2−^ and OH^-^), which might be released from the dissolved biochars and contribute to the Pb(II) precipitation process. In another study, *Ganoderma lucidum* derived biochars were significantly influenced by the pyrolysis temperature [100]. A reduction in biochar yield containing O/C, H/C and O functional groups and increased biochar ash, thermal stability and the specific surface were observed with temperature increase (250–650 °C). The high-temperature biochar can be an excellent adsorbent for heavy metal removal due to its wide specific surface area and mesoporous structure. Zhang et al. [101] utilized *Agaricus bisporus* substrate-derived biochar and used it to remove cadmium, copper and zinc from an aqueous solution. They concluded that the removal mechanism using biochar produced at 350 °C was primarily via ion exchange. In contrast, biochar was produced at a moderate temperature of 550 °C mainly via coordination with π electrons and mineral precipitation.

## 8. Edible Mushrooms Derived Nanoparticles (NPs)

The high concentrations of extracellular enzymes serve as bio-reducing and stabilizing agents for NP synthesis. NPs made from mushrooms are of better quality than those made from bacteria. Metal NPs synthesized using constituents such as enzymes and metabolites secreted by mushroom cells reduce the toxicity of substances [105,106]. The use of NPs is rising, especially in biomedicine and pharmaceuticals, because of their unique physicochemical properties. In the bottom-up approach, biogenic NPs are synthesized, resulting in atoms/compounds that act as the building blocks and possess the ability to self-assemble to form the final product [44]. Numerous metal oxide/noble metal NPs have been developed using extracts of edible mushrooms, as listed in Table 7.

CuNPs derived from an aqueous extract of *Agaricus bisporus* showed the highest inhibitory activity against *Enterobacter aerogens* with an inhibition zone of 15.00 mm [107]. Antioxidant assays showed a possible scavenging effect against the ABTS radical (82%), the NO radical (76%) and the DPPH radical (72%). AgNPs derived using *Ganoderma lucidum* extract showed antibacterial effects with minimum inhibitory concentration (MIC) values of 128, 16 and 64 μg L^−1^ against Gram-positive bacteria (*B. cereus*, *E. hirae*, *S. aureus*) and 16, 64 and 128 μg L^−1^ against Gram-negative bacteria (*L. pneumophila* subsp. *pneumophila*, *E. coli*, *P. aeruginosa*). AgNPs derived from the pine mushroom (*Tricholoma matsutake*) displayed a zone of inhibition of 21.00mm against *E. coli* [115]. The anti-cancerous effect of *Ganoderma neo-japonicum* derived AgNPs against MDA-MB-231 human breast cancer cells was dose-dependent, according to Gurunathan et al. [118], and cells exposed to AgNPs generated more reactive oxygen species and hydroxyl radicals. At dilutions ranging from 10 to 30 g mL^−1^, AuNPs derived from *Pleurotus florida* extract by photo-irradiation demonstrated dose-dependent antiproliferative activity against various cancer cell lines (A-549, K-562, HeLa and MDA-MB) [109]. Chaturvedi et al. [110] investigated cytotoxicity and observed that AgNPs and AuNPs formed by *Pleurotus sajor-caju* extract (PS) were effective against the HCT-116 cancer cell line. HCT-116 cancer cell viability was inhibited by *P. sajor-caju* extract, AuNPs and AgNPs, with IC_50_ values of 60, 80 and 50 g mL^−1^, respectively. Compared to other PS extracts and AuNPs, green synthesized AgNPs showed strong antiproliferative activity due to ROS generation, which led to oxidative stress and resulted in improper protein functionality and integrity.

## 9. Edible Mushrooms Derived Carbon Dots

Carbon dots (CDs), photoluminescent substances with a size of less than 10 nm, can be synthesized by top-down and bottom-up approaches [44]. The top-down synthetic route involves a complex and synthetic condition; a broad carbon structure is broken down using electro-oxidation, acid-assisted chemical oxidation, and laser ablation [44]. However, the bottom-up approach, which relies on plants and their by-products instead of the chemicals, is superior compared to the top-down approach. Proteins, carbohydrates, lipids, lignin and cellulose are all abundant in biological materials. Edible mushrooms are relatively inexpensive and contain various chemical constituents such as carbon, oxygen, phosphorus and nitrogen, often depicted as carboxyl and amine groups. The presence of carbohydrates, amino acids, polysaccharides, citric acid, flavonoids, lipids, vitamins and proteins make them ideal for CDs development [119]. CDs have also shown effectiveness in biomedical applications and energy storage systems, including water purification, pathogen identification, environmental research and heavy metal and additive detection in food (Table 8).

*Pleurotus* sp. derived CDs were prepared by Boobalan et al. [119], and the authors found that the presence of hydroxyl and carboxyl groups (derived from amino acids and polysaccharides) caused them to bind with selective Pb^2+^ ions, forming lead hydroxide and carboxylate, which causes their quenching. Further, they observed an increase in antibacterial activity with increased CDs concentration, and they reported anti-cancer activity with a half-maximal inhibitory concentration (IC_50_) of 3.34 g mL^−1^. In addition to this, Zulfajri et al. [121] developed CDs using oyster mushroom (OM) for sensing nitroarenes (NAs). The study demonstrated that the sensing mechanisms such as fluorescence resonance energy transfer (FRET), inner filter effect (IFE) and photo-induced electron transfer (PET) quenched the fluorescence emission intensity of OM-CDs by NAs. The spiked underground water recoveries were found to be 97.20 and 102.60%, with RSDs under 2%, representing the reliability of OM-CDs as a sensor for NAs with good repeatability, precision and recovery. Yang et al. [123] developed CDs that can detect hyaluronic acid in human urine samples in concentrations ranging from 50 pM to 50 mM.

## 10. Edible Mushrooms Based Skin Care Formulations

Cosmetics are personal care products that are used to cleanse and beautify the skin [124]. The demand for cosmetics containing natural ingredients is increasing due to their organic, healthier and environmentally friendly characteristics [125]. Lentinan, carotenoids, ceramides, schizophyllan and ω-3, ω-6 and ω-9 fatty acids as well as resveratrol obtained from macro fungi, especially mushrooms, are now paving their way into cosmetics [126,127]. These are reported to treat beauty issues such as fine lines, wrinkles, uneven tone and texture due to the antioxidant and anti-inflammatory traits. There are few studies where edible mushrooms are used in skincare formulations, as compiled in Table 9.

In a study conducted by Taofiq et al. [129], terpenoids (ganoderic acids C2, A and H) and phenolics (protocatechuic, p-hydroxybenzoic and syringic acids) were identified as essential cosmeceutical components in the *G. lucidum* extracts. Further, Taofiq et al. [133] reported that ethanolic extracts of *Agaricus bisporus* and *Pleurotus ostreatus* contain essential bioactive compounds, but they readily are degraded and oxidized. Thus, they developed microencapsulated extracts incorporated into a semi-solid base cream. Their efficacy was compared with those of free-forms in terms of bioactivity, in vitro release and real-time conditions (up to 6 months). Antimicrobial and anti-tyrosinase activities were also observed for the formulations prepared with the encapsulated forms, but the extract released over time was found to be insufficient to exert an impact on the antioxidant action. Lourith et al. [132] developed moisturizing hand sanitizer using snow mushroom polysaccharides. The sanitizer showed no signs of irritation when applied to the volunteers and significantly (*p* < 0.05) moistened the skin compared with the placebo.

## 11. Conclusions

Edible mushrooms and their by-products are extensively involved in different fields with diverse applications. Due to their nutritional and functional values, mushrooms are taken as dietary supplements with probiotics and are fortified as RTE and RTC food. The bioactive molecules obtained from them are found to have applications in food products. Other than this, mushrooms are found to play an important role in the production of biochar, biosorbent, carbon dots, media, nanoparticles and skincare formulations. All the above-stated products synthesized or developed with the aid of mushrooms have shown effective results in invitro studies. However, edible films/coatings and skincare formulations developed from these edible mushrooms are still limited to the invitro level and are not yet being exploited at the commercial and industrial levels. Additionally, these edible mushrooms and their waste have immeasurable economic potential in different industries, and it can often lead to the synthesis of novel products. Still, these edible mushrooms remain an untapped resource with vast industrial applications. Thus, there is a dire need for sensible and responsible management within the production system to explore the potential of these edible mushrooms.

## Figures and Tables

**Table 1:** Mushrooms fortified in ready-to-eat (RTE) and ready-to-cook (RTC) foods.

Edible Mushroom Common Name	Scientific Name	Food Product	Beneficial Effects	Reference
Milky white	*Calocybe indica*	Cookies	Increase in protein, fiber, minerals and β-glucan, phenolic, flavonoids and antioxidants; decrease in starch, reduction in glycemic index	[17]
Oyster	*Pleurotus sajor-caju*	Biscuits	Increase in concentration of protein, dietary fiber, ash and reduction in carbohydrate	[19]
Shiitake	*Lentinula edodes*	Chips	Improvement in quality attributes (color, sensory evaluation)	[20]
Oyster	*Pleurotus ostreatus*	Biscuits	Enhancement of nutritional quality	[21]
White button	*Agaricus bisporus*	Ketchup	Increase in ash content, crude fiber, protein, total soluble solids, and reducing sugars; decrease in total sugars	[22]
Oyster	*Pleurotus ostreatus*	Jam	Increase in total soluble solids, percent acidity and reducing sugar, decrease in pH and non-reducing sugar	[23]
White button	*Agaricus bisporus*	Mushroom tikki and stuffed mushroom	Increase in protein, dietary fiber, antioxidant and phenolic components	[24]
Oyster	*Pleurotus ostreatus*	Soup	Increase in nutritional value	[25]
Chestnut	*Agrocybe aegerita*	Snacks	Manipulation of glycemic response of individuals	[26]
Oyster	*Pleurotus sajur-caju*	Biscuit	Increase in the mineral content	[27]
Oyster	*Pleurotus ostreatus*	Vegetable mixture diets	Highly acceptable, nutritious, delicious, ready-to-eat diet	[14]
Oyster	*Pleurotus ostreatus*	Processed cheese spreads	High moisture, ash and protein content, total viable counts and spore former bacteria was lower in processed cheese supplemented with mushrooms	[28]
Oyster	*Pleurotus ostreatus*	Biscuit	Higher moisture, protein, ash content, higher hardness, darker and redder in color	[29]
Oyster	*Pleurotus ostreatus*	Spreadable processed cheese	Increase in total solids, protein, fibers and carbohydrates	[30]
Oyster	*Pleurotus sajor-caju*	chicken patty	Reduction in fat content, no change in protein and β-glucan	[31]
White button	*Agaricus bisporus*	Pasta	Improved antioxidant activity, increase moisture content, carbohydrates, decreased crude fiber, crude protein, and fat	[32]
Oyster	*Pleurotus sajor-caju*	Cookies	High protein content, low-fat content, high fiber, minerals and vitamin content	[33]
White button	*Agaricus bisporus*	Pasta	Decrease in the extent of starch degradation, increase in total phenolic content and antioxidant capacities	[34]
White jelly	*Tremella fuciformis*	Patty	Oil holding capacity of mushroom has a positive effect on cooking yield of patty as well as senses	[35]
Oyster	*Pleurotus ostreatus*	Instant noodles	Increase in protein and fiber content	[36]
White button	*Agaricus bisporus*	Beef burgers	Reduction in the fat content of beef burgers	[37]
Oyster	*Pleurotus ostreatus*	Instant soup premix	Rich in protein, crude fiber, minerals and low in fat, carbohydrate and energy value	[38]
White button	*Agaricus bisporus*	Sponge cake	Increase in apparent viscosity, volume, springiness and cohesiveness values	[39]
Oyster	*Pleurotus sajor-caju*	Biscuit	Reduction in starch pasting viscosities, starch gelatinization enthalpy value, increases in protein, crude fiber and mineral content	[16]
Shiitake	*Lentinula edodes*	Noodles	Improvement in nutritional profile and reduction in the glycemic index of foods	[18]
King tuber	*Pleurotus tuber-regium*	Cookies	Higher protein, ash, crude fiber, water-soluble vitamins and minerals	[40]
Oyster	*Pleurotus ostreatus*	Noodles	Lower level of carbohydrate, fat, and sodium	[41]
King trumpet	*Pleurotus eryngii*	Sponge cake	Increase in ash and proteincontent	[42]
White button, Shitake, Porcini	*Agaricus bisporus, Lentinula edodes, Boletus edulis*	Pasta	High firmness and tensile strength	[43]

**Table 2:** Mushrooms and their residue-based edible film/coatings.

Edible Mushrooms Common Name	Scientific Name	Product Used	Compounds	Key Findings	References
White button	*Agaricus bisporus*	Fruit bars	Chitosan	Increased antioxidant capacity, ascorbic acid content, fungal growth prevention and firmness	[45]
White button	*Agaricus bisporus*	Fresh-cut melons	Chitosan	Enhance fruit firmness, inhibit off-flavors and reduce the microbial counts (up to 4 log CFU g^−1^).	[47]
Velvet shank	*Flammulina velutipes*	ND	Polysaccharide	High tensile strength, barrier property to water vapor and oxygen	[46]
Shiitake, Velvet shank	*Lentinula edodes, Flammulina velutipes*	ND	Insoluble dietary fibers	Highest tensile strength and an effective barrier to water vapor	[48]
Indian oyster	*Pleurotus pulmonarius*	ND	Flour	Significant barrier properties and mechanical strength	[49]

ND—not defined; CFU—colony-forming unit.

**Table 3:** Applications of mushrooms as prebiotics.

Edible Mushrooms Common Name	Scientific Name	Probiotic Used	Form of Mushroom Used	Applications	References
White button	*Agaricus bisporus*	Probiotics mixture (Protexin 6 × 10^7^CFU gm^−1^)	Powder	Lowered total cholesterol, LDL cholesterol, triglyceride concentrations, oxidative stress and dyslipidemia in hypercholesterolemic rats	[50]
Wood ear/Jew’s ear	*Auricularia auricula*	*Lactobacillus acidophilus* La-5, *Bifidobacterium bifidum* Bb-12	Extract	Enhancement in the survival rate of probiotics toabout 0.43 and 0.51 log CFU g^−1^; improved probiotic protection and functional properties of symbiotic yogurt	[55]
White button	*Agaricus bisporus*	*Saccharomyces cerevisiae*	Powder	Improvement in the meat quality with the incorporation of mushroom and probiotics in the broiler diet	[56]
Oyster	*Pleurotus sajor-caju*	*Lactobacillus fermentum* OVL	Powder	Increase in neutrophil count in rats, decrease in lymphocyte count	[57]
Oyster	*Pleurotus ostreatus*	PrimaLac (*Lactobacillus acidophilus, Lactobacillus casei, Bifidobacterium bifidium, Enterococcus faecium*)	Powder	Decrease in abdominal fat on the carcass, increase in HDL concentration in plasma	[53]
Caterpillar	*Cordyceps militaris*	*Lactobacillus plantarum*	Spent mushroom substrate	Increase in the specific growth rate, weight gain, final weight in fish fed supplemented diets	[58]
Shiitake	*Lentinus edodes*	1.0 ×10^8^ CFU g^−1^(*Lactobacillus acidophilus*, *Lactobacillus casei*, *Bifidobacterium bifdium*, *Enterococcus faecium*)	Extract	No weight gain in broiler chickens	[59]
*King oyster*	*Pleurotus eryngii*	*Lactobacillus plantarum*	Powder	Growth stimulation, immunity and disease resistance	[52]

LDL—low-density lipoproteins; HDL—high density lipoproteins.

**Table 4:** Mushrooms and their residue-based media.

Edible Mushrooms Common Name	Scientific Name	Media Composition	Purpose/Utilization	References
Velvet shank	*Flammulina velutipes*	Spent mushroom substrate, perlite, and vermiculite	Growing media for tomato and cucumber seedlings	[66]
White button, Oyster	*Agaricus bisporus, Pleurotus ostreatus*	Spent mushroom substrate, and Sphagnum peat	Growing media for tomato, courgette and pepper	[67]
Velvet shank	*Flammulina velutipes*	Spent mushroom substrate, and chicken manure compost	Growing media for honeydew melon	[68]
Velvet shank	*Flammulina velutipes*	Spent mushroom substrate, calcium carbonate, wheat bran, and yeast extract and inorganic salts	Production media for *Bacillus thuringiensis*	[69]
Oyster	*Pleurotusf lorida*	Spent mushroom substrate	Production media for lignocellulolytic enzymes	[70]
Oyster	*Pleurotus ostreatus*	Spent mushroom substrate	Production media for *Lactococcus lactis*	[71]
Oyster	*Pleurotus ostreatus*	Spent mushroom substrate, paddy straw, and soybean cake	Biopesticide (*Trichoderma asperellum*) development	[72]
ND	ND	Spent mushroom substrate and peat moss	Growing media for Chinese kale	[73]
ND	ND	Spent mushroom substrate, perlite, and vermiculite	Growing media for lettuce seedlings	[74]
ND	ND	Spent mushroom substrate, polished rice, full-fat soybean, and rice bran	Production media for arachidonic acid by *Mortierella* sp.	[75]
ND	ND	Spent mushroom substrate, and poultry cooked bones	Production media for solubilizationphosphate by *Bacillus megaterium*	[76]

ND—not defined.

**Table 5:** Mushroom-derived biosorbents and their applications.

Edible Mushrooms Common Name	Scientific Name	Drying Temperature/Time	Applications	References
Oyster	*Pleurotus florida*	RT/24 h	Showed 100% removal of Fe^2+^ from the water sample	[79]
White button	*Agaricus bisporus*	80 °C/24 h	Successfully biosorbed Reactive Blue 49 dye (1.85 × 10^−4^ mol g^−1^) from water	[80]
Oyster	*Pleurotus ostreatus*	40 °C/24 h	Showed greater adsorption against Pb^2+^(85.91 mg g^−1^) in water	[81]
Oyster	*Pleurotus ostreatus*	60 °C/24 h	Biosorbed 3.8 mg g^−1^ of Cd^2+^	[82]
Oyster, Black morels	*Pleurotus ostreatus*, *Morchella conica*	RT/4 days	Adsorbed methylene blue (82.81 and 38.47 mg g^−1^) and for malachite green (64.13 and 39.28 mg g^−1^)	[83]
Velvet shank	*Flammulina velutipes*	60 °C/24 h	Maximum removal capacity against copper ions was 15.56 mg g^−1^	[84]
Shiitake	*Lentinula edodes*	Freeze-dried/24 h	Maximum absorption against Congo red was 217.86 mg g^−1^	[85]
Oyster	*Pleurotus ostreatus*	78 °C/48 h	Showed maximum biosorption against uranium ion (19.95 mg g^−1^)	[86]
Oyster	*Pleurotus ostreatus*	80 °C/ND	Showed maximum biosorption against Ni^2+^ (20.71 mg g^−1^)	[87]
King trumpet	*Pleurotus eryngii*	60 °C/24 h	Showed maximum biosorption against Pb^2+^ (3.30 mg g^−1^)	[88]
Lingzhi	*Ganoderma lucidum*	60 °C/72 h	Maximum biosorption against malachite green (40.65 mg g^−1^), safranine T (33.00 mg g^−1^), and methylene (22.37 mg g^−1^)	[89]
King trumpet	*Pleurotus eryngii*	60 °C/24 h	Removed 88.38% of NO_3_^−^	[90]

RT—room temperature; ND—not defined.

**Table 6:** Applications of biochar derived from mushrooms and their residues.

Edible Mushrooms Common Name	Scientific Name	Process and Conditions Required for Biochar Formation	Applications	References
Oyster, Shiitake	*Pleurotus ostreatus*, *Lentinula edodes*	Pyrolysis at 700 °C for 2 h	Adsorbed 326mg g^−1^ and 398mg g^−1^ of lead Pb(II) from the water	[99]
Lingzhi	*Ganoderma lucidum*	Pyrolysis at 650 °C for 2 h	Showed maximal adsorption against Pb2^+^ (262.76 mg g^−1^) and Cd^2+^ (75.82 mg g^−1^)	[100]
White button	*Agaricus bisporus*	Pyrolysis at 750 °C for 3 h	Showed maximal adsorption against Cu^2+^(65.2 mg g^−1^), Cd^2+^(76.3 mg g^−1^), and Zn^2+^(44.4 mg g^−1^) in water	[101]
ND	ND	Pyrolysis at 300 °C for 90 min	Showed maximal adsorption against Pb2^+^ (21.0 mg g^−1^), Cu^2+^(18.8 mg g^−1^), Cd^2+^(11.2 mg g^−1^) and Ni^2+^(9.8 mg g^−1^) in water	[102]
ND	ND	Pyrolysis at 450 °C for 4 h	Showed maximal adsorption against crystal violet (1057mg g^−1^) in wastewater	[103]
ND	ND	Pyrolysis at 500 °C for 2 h	Showed maximal adsorption against fluoride (36.5 mg g^−1^) in water	[104]

ND—not defined.

**Table 7:** Mushroom-derived nanoparticles and their applications.

Edible Mushrooms Common Name	Scientific Name	Types of Nanoparticles Synthesized	Reaction Temperature/Time	Morphology	Size	Applications	References
White button	*Agaricus bisporus*	Copper	RT/24 h	Spherical	2–10 nm	Antibacterial activity against *Enterobacter aerogens*; Antioxidant activity using DPPH, and ABTS; Anti-cancer activity against cancer cell lines SW620 (colon cancer)	[107]
Brown oyster	*Pleurotus cystidiosus*	Gold	29 °C/24 h	ND	ND	Antioxidant activity using DPPH, and ABTS	[108]
Oyster	*Pleurotus florida*	Gold	70 °C/1.5 h	Spherical	2–14 nm	Anti-cancer activity against cancer cell lines A-549 (Human lung carcinoma), K-562 (Human chronic myelogenous leukemia bone marrow), HeLa (Human cervix) and MDA-MB (Human adenocarcinoma mammary gland)	[109]
Oyster	*Pleurotus ostreatus*	Gold	29 °C/24 h	Spherical	22.9 nm	Antioxidant activity using DPPH, and ABTS	[108]
Oyster	*Pleurotus sajor-caju*	Gold	RT/12 h	Spherical	16–18 nm	Anti-cancer activity against cancer cell lines HCT-116 (colon cancer)	[110]
King tuber	*Pleurotus tuber-regium*	Selenium	RT/24 h	Spherical	91–102 nm	Anti-cancer activity against gastric adenocarcinoma AGS	[111]
Oyster	*Pleurotus ostreatus*	Silver	25 °C/48 h	Spherical	17.5 nm	Anti-cancer activity against cancer cell lines HepG2 (human liver) and MCF-7 (breast)	[112]
Lingzhi	*Ganoderma lucidum*	Silver	ND/ND	Spherical	15–22 nm	Antioxidant activity using DPPH; Antibacterial activity against *Staphylococcus aureus,* *Enterococcus hirae, Bacillus cereus*, *Escherichia coli, Pseudomonas aeruginosa,* *Legionella pneumophila* subsp. *Pneumophila*; and antifungal activity against *Candida albicans*	[113]
Matsutake	*Tricholoma matsutake*	Silver	RT/30 min	Spherical	10–70 nm	Antibacterial activity against *Bacillus cereus*, *Escherichia coli*	[114]
Milky white, Oyster, White button, Lingzhi	*Calocybe indica*, *Pleurotus ostreatus*, *Agaricu sbisporus*, *Ganoderma lucidum*	Silver	RT/12 h	Spherical	80–100 nm	Antibacterial activity against *Staphylococcus aureus*	[115]
Pink oyster	*Pleurotus djamor*	Titanium oxide	RT/20 min	Spherical	31 nm	Antibacterial activity against *Corynebacterium diphtheria*, *Pseudomonas fluorescens*, and *Staphylococcus aureus*; Anti-cancer activity against cancer cell lines A-549 (Human lung carcinoma); larval toxicity against *Aedes aegypti*, *Culex quinquefasciatus*	[116]
Pink oyster	*Pleurotus djamor*	Zinc oxide	RT/24 h	Sphere	74.36 nm	Antioxidant activity using DPPH, ABTS, and H_2_O_2_; larval toxicity against *Aedes aegypti*, *Culex quinquefasciatus*; Antibacterial activity against *Corynebacterium diphtheria*, *Pseudomonas fluorescens*, and *Staphylococcus aureus*	[117]

RT—room temperature; ND—not defined; DPPH-2,2-diphenyl-1-picrylhydrazyl-hydrate; ABTS-2,2’-azino-bis(3-ethylbenzothiazoline-6-sulfonic acid).

**Table 8:** Mushrooms as a carbon source for preparing carbon dots.

Edible Mushrooms Common Name	Scientific Name	Production Conditions	Applications	References
Oyster	*Pleurotus* sp.	Hydrothermal/120 °C/4 h	Selective sensitivity for Pb^2+^; Antibacterial activity against *Staphylococcus aureus, Klebsiella pneumoniae and Pseudomonas aeruginosa*; Anti-cancer activity against breast cancer cells (MDA-MB-231)	[119]
Velvet shank	*Flammulina velutipes*	Hydrothermal/250 °C/4 h	Sensed Cr^6+^ with a limit of detection 0.73 µM and volatile organic compounds	[120]
Oyster	*Pleurotu* ssp.	Hydrothermal/200 °C/25 h	Sensed nitroarenes in water samples	[121]
Paddy straw	*Volvariella volvacea*	Hydrothermal/200 °C/25 h	Sensed Pb^2^ with limit of detection 12 nM and for Fe^3+^ 16 nM	[122]
ND	ND	Hydrothermal/200 °C/6 h	Sensed hyaluronic acid and hyaluronidase	[123]

ND—not defined.

**Table 9:** Mushroom-based skincare formulations.

Edible Mushrooms Common Name	Scientific Name	Product Base	Applications	References
White button, Oyster, Shiitake	*Agaricus bisporus*, *Pleurotus ostreatus*, *Lentinula edodes*	Cream	Anti-inflammatory; anti-tyrosinase; antioxidant and antibacterial activity	[128]
Lingzhi	*Ganoderma lucidum*	Cream	Anti-tyrosinase; antioxidant and antibacterial activity	[129]
Oyster	*Pleurotus ostreatus*	Cream	Skin fairness	[130]
Oyster	*Pleurotus ostreatus*	Gel	Anti-tyrosinase; antioxidant activity	[131]
Snow	*Tremella fuciformis*	Gel	Hand sanitizer	[132]
White button, Oyster	*Agaricus bisporus*, *Pleurotus ostreatus*	Cream	Anti-tyrosinase; antioxidant and antibacterial activity	[133]

## Data Availability

Not applicable.
